# Phylogeography of Supralittoral Rocky Intertidal *Ligia* Isopods in the Pacific Region from Central California to Central Mexico

**DOI:** 10.1371/journal.pone.0011633

**Published:** 2010-07-21

**Authors:** Luis A. Hurtado, Mariana Mateos, Carlos A. Santamaria

**Affiliations:** Department of Wildlife and Fisheries Sciences, Texas A&M University, College Station, Texas, United States of America; University of Canterbury, New Zealand

## Abstract

**Background:**

*Ligia* isopods are widely distributed in the Pacific rocky intertidal shores from central California to central Mexico, including the Gulf of California. Yet, their biological characteristics restrict them to complete their life cycles in a very narrow range of the rocky intertidal supralittoral. Herein, we examine phylogeographic patterns of *Ligia* isopods from 122 localities between central California and central Mexico. We expect to find high levels of allopatric diversity. In addition, we expect the phylogeographic patterns to show signatures of past vicariant events that occurred in this geologically dynamic region.

**Methodology/Principal Findings:**

We sequenced two mitochondrial genes (Cytochrome Oxidase I and 16S ribosomal DNA). We conducted Maximum Likelihood and Bayesian phylogenetic analyses. We found many divergent clades that, in general, group according to geography. Some of the most striking features of the *Ligia* phylogeographic pattern include: (1) deep mid-peninsular phylogeographic breaks on the Pacific and Gulf sides of Baja peninsula; (2) within the Gulf lineages, the northern peninsula is most closely related to the northern mainland, while the southern peninsula is most closely related to the central-southern mainland; and, (3) the southernmost portion of the peninsula (Cape Region) is most closely related to the southernmost portion of mainland.

**Conclusions/Significance:**

Our results shed light on the phylogenetic relationships of *Ligia* populations in the study area. This study probably represents the finest-scale phylogeographic examination for any organism to date in this region. Presence of highly divergent lineages suggests multiple *Ligia* species exist in this region. The phylogeographic patterns of *Ligia* in the Gulf of California and Baja peninsula are incongruent with a widely accepted vicariant scenario among phylogeographers, but consistent with aspects of alternative geological hypotheses and phylo- and biogeographic patterns of several other taxa. Our findings contribute to the ongoing debate regarding the geological origin of this important biogeographic region.

## Introduction

Organisms restricted to specific patchy habitats and with extremely low vagility represent promising models for biodiversity and phylogeographic studies. Genetic characterization of their populations can reveal unexpected levels of previously unknown biodiversity [e.g. 1],[2],[3]. Furthermore, their phylogeographic patterns may offer clues to past geological and environmental events, which can improve our understanding on the biogeographic history of a region [e.g. 4],[5]–[7]. Coastal isopods of the genus *Ligia* exemplify an organism with an apparently very limited vagility and high restriction to a patchy habitat, yet wide geographic distribution. Thus, they have the potential for revealing high levels of cryptic biodiversity and for serving as biogeographic indicators.

*Ligia* is grouped within the Oniscidea, a group that includes all terrestrial isopods, but evolved from a marine ancestor [8]. *Ligia* has a worldwide distribution and currently includes over 30 nominal species, most of which are halophilic forms occurring in the supralittoral zone of rocky shores worldwide [9], [10]. However, approximately seven species are terrestrial and occur in montane habitats of tropical regions [11]. *Ligia* exhibits morphological, physiological and behavioral characteristics that are intermediate between ancestral marine and fully terrestrial isopods [12].

Coastal *Ligia*, also known as rock lice or sea slaters, are found in a very narrow vertical range of the rocky intertidal supralittoral. Low desiccation resistance and a primarily algal detritus diet constrain them to the dry and splash zones of the upper rocky intertidal; where they can take up water from droplets and puddles by capillarity and from water vapor directly from the air, and hide under rocks and in crevices to minimize water loss and hide from predators [12]. They are well adapted for terrestrial locomotion on rocky beaches, but not on sandy beaches, where the lack of shelter also makes them more vulnerable to predators and desiccation. Although they retain the ancestral ability for underwater gas exchange, they actively avoid entering the water, except when escaping from predators or by accidental wave dislodgement. Underwater locomotion is used to regain the shore after such events. However, the potential for active long-distance dispersal and predator avoidance underwater is extremely limited [13]. Their long-distance dispersal capabilities are further limited by the fact that they are direct developers (i.e., lack a planktonic larval stage). Females carry tens of eggs in a thoracic pouch or marsupium, in which offspring develop from fertilization to a juvenile stage [8]. Adults range in size from 2–8 cm. Because of the above characteristics, *Ligia* isopods appear to be highly constrained throughout their life cycle to the same rocky beach, since they do not actively disperse in the water at any stage, and large areas of sandy beaches, estuaries, and cliffs, separating discrete rocky beaches may constitute effective dispersal barriers. Phylogeographic studies of *Ligia* isopods in different parts of the world have revealed high levels of allopatric differentiation, consistent with expectations from their life history [11], [14].

Only one *Ligia* species, *Ligia occidentalis*, is usually recognized between southern California and central Mexico, including the Gulf of California; and in central California, *L. occidentalis* overlaps with *L. pallasi* [15]. However, some authors suggest the possibility of multiple *Ligia* species in southern California and the Gulf of California [16], [17]. Rocky intertidal communities of this region are extremely biodiverse [17]–[19]; and given the biology of *Ligia*, phylogeographic studies of this isopod are likely to reveal high levels of cryptic biodiversity. Furthermore, *Ligia* has a high potential for preserving in its genealogy, signatures of past vicariant events in this tectonically dynamic region [20]. Thus, phylogeographic studies of *Ligia* in California and western Mexico may contribute to understanding highly controversial aspects of the geological history of this region.

**Figure 1:**
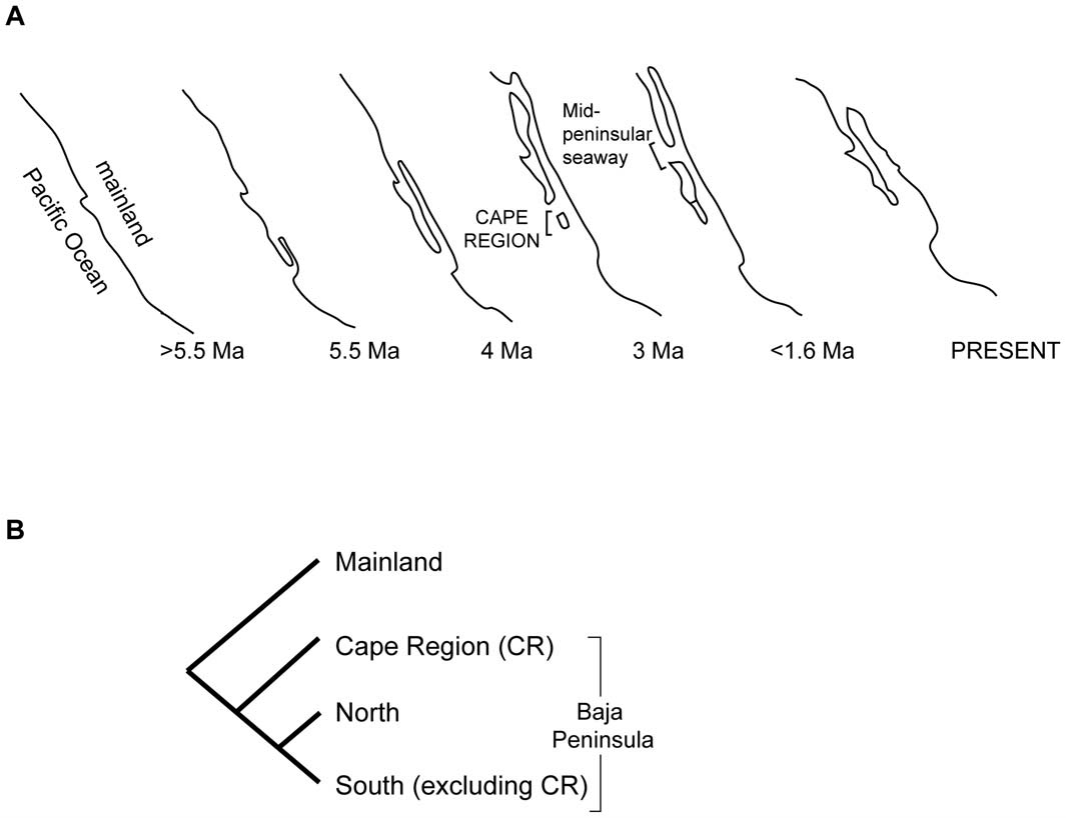
Vicariant hypothesis for the origin of the Gulf of California and Baja Peninsula described in Riddle [**22**]. **A.** Sequence of geological events. **B.** Expected phylogeny of an organism showing signatures of these vicariant events [modified from 22].

For example, the origin of the Gulf of California and the Baja Peninsula has been subject to different interpretations [e.g. 21],[22]–[27]. Phylogeographic studies of this region, however, have traditionally followed a geological framework described in Riddle et al. [22] (Figure 1a). Accordingly, four main vicariant events occurred during the history of this region: (1) A small peninsula separated the southern portion of the Gulf from the Pacific Ocean ∼5.5 million years ago (Ma), when the Gulf began to form, and a subsequent northern extension separated the rest of the peninsula from mainland ∼4 Ma [23]; (2) northward transgressions of the Gulf into low-lying deserts in southern California and Arizona occurred, and isolated the whole Baja from the mainland ∼3 Ma [21]; (3) a trans-peninsular seaway isolated the Baja Cape region (i.e., the southern tip of Baja) from the rest of Baja ∼3 Ma [21], [23]; and (4) a trans-peninsular seaway across the mid-peninsula isolated northern and southern peninsular biotas ∼1 Ma [28]. Figure 1b shows the expected phylogeny for an organism depicting signatures of the above vicariant events. However, alternative geological hypotheses exist, which consider a much earlier origin of the Gulf and that its formation proceeded from north to south [25], [26], [29] (addressed in Discussion section).

Herein, we analyzed the phylogeographic patterns of *Ligia* from central California to central Mexico. We discover remarkable levels of cryptic genetic diversity in this isopod and discuss the phylogeographic patterns in light of the vicariant hypotheses that have been proposed for this region.

## Materials and Methods

**Figure 2 pone-0011633-g002:**
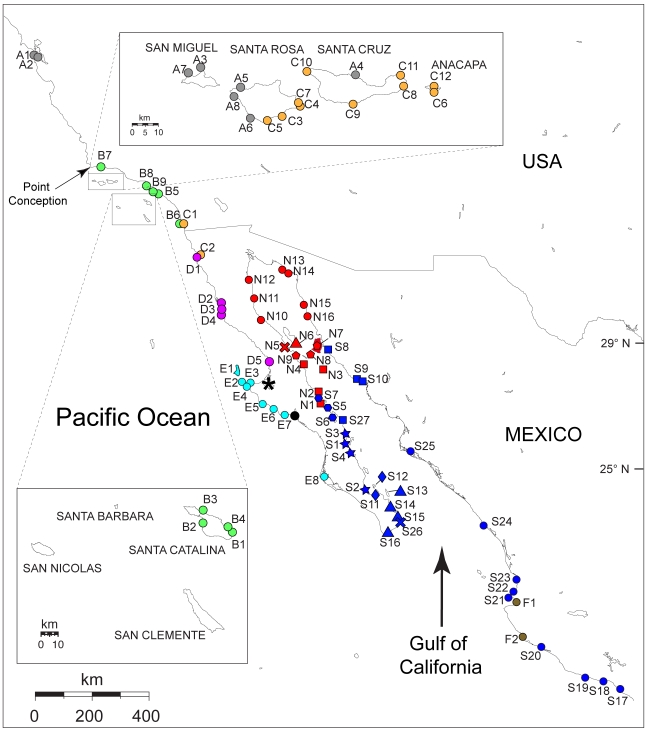
Sampled localities. Color and shape correspond to clades in Figs. 3 and 4. A1- Princeton; A2-Coyote Point; A3-Harris Point; A4- Orizaba; A5-NW Talcott; A6-China Point; A7-Otter Harbor; A8-Fossil Reef; B1-Industrial Area; B2-Little Harbor; B3-Ithsmus Cove; B4-Descanso; B5-Long Beach; B6-San Diego; B7-Refugio; B8-Malaga Cove; B9-Cabrillo; C1-San Diego; C2-Corona Ensenada; C3- Ford Point; C4-East Point; C5-Johnsons Lee; C6-Side Frenchy's; C7-Sandy Beach; C8-Smugglers Cove; C9-Willows; C10-Fraser Cove; C11-Scorpion; C12-Frenchys; D1-Bufadora Ensenada; D2-San Quintin; D3-Punta Baja; D4-Arroyo Ancho; D5-Tomatal; E1-Cedros Is. (2 localities); E2-Punta Eugenia; E3- El Chevo, El Queen, and Malarrimo; E4-Tortugas; E5-Bahía Asuncion; E6-San Hipolito; E7-Punta Abreojos; E8-Puerto San Carlos; F1-Vallarta; F2-Careyes; S1-Loreto; S2-Cajete, San Evaristo; S3-San Brunito; S4-San Cosme; S5-Punta Chivato and Mulege; S6- Bahía Concepción: Bahía Concepcion N, Punta Sueño, Buenaventura, Requeson, Bahía Armenta, Bahía Concepcion S; S7-San Lucas and Santa Rosalia; S8-Bahía Kino; S9-San Carlos; S10-Guaymas; S11-La Paz; S12-IslaEspiritu Santo (2 localities) and Isla Partida; S13-Isla Cerralvo (3 localities); S14-Ensenda de los Muertos and Barriles; S15-Frailes; S16-Cabo San Lucas and El Arco; S17-Barra Potosi; S18-Ixtapa, Zihuatanejo; S19-Carrizalillo; S20-Manzanillo and Boquita; S21-Punta Mita; S22-Isla Coral; S23-San Blas, Platanitos, and Aticama; S24-Mazatlan (2 localities); S25-Topolobampo; S26-Cabo Pulmo; S27-San Nicolas; N1-San Bruno; N2-Santa Rosalia; N3-Isla San Pedro Martir; N4-San Francisquito; N5-Bahía de los Angeles; N6-Isla Angel de la Guarda (Viborita); N7-Isla Tiburon (3 localities), Isla Cholludo, Isla Datil; N8-Isla San Esteban; N9-San Rafael; N10-San Luis Gonzaga; N11-Puertecitos; N12-San Felipe; N13-La Cholla; N14 Puerto Peñasco; N15-Puerto Lobos; N16-Puerto Libertad. * denotes Guerrero Negro Lagoon; black circle denotes San Ignacio Lagoon.

Samples were collected during 2007–2009 by hand on rocky intertidal shores from 122 localities distributed from central California to central Mexico (Fig. 2; Supplemental Table S1). A preliminary examination of DNA sequences of the mitochondrial Cytochrome Oxidase I gene (COI) for a subset of >40 localities from the Gulf of California and Baja Pacific that included multiple individuals per locality, revealed that most localities do not share haplotypes (Hurtado et al. unpublished). The only instances of haplotype sharing occurred among a few neighboring localities that probably represent the same population (results not shown). Thus, to understand phylogeographic patterns at our geographic scale of interest, we focused on maximizing the number of localities and included one individual per locality.

We extracted DNA from leg segments using the DNEasy kit (Qiagen, Inc., Valencia, CA). For all individuals, we performed PCR amplification of two mitochondrial gene fragments using published primers and PCR conditions: a 710-bp region of COI [30]; and, a ∼520-bp region of the mitochondrial 16S rDNA gene [primers 16Sar/16Sbr; 31]. PCR products were cleaned with Exonuclease and Shrimp Alkaline Phosphatase and cycle sequenced with the BigDye® Terminator v3.1 Cycle Sequencing Kit (Applied BioSystems, Foster City, CA). Sequenced products were cleaned with Sephadex® G-50 (Sigma-Aldrich, St. Louis, MO) and run on a 3100 Genetic Analyzer. We used Sequencher 4.8 (Gene Codes, Ann Arbor, MI) for sequence editing and primer removal. None of the COI sequences had premature stop codons or frame shifts, suggesting that they are not pseudogenes. The 16S rDNA sequences were aligned with Clustal×2.0 [32] and edited manually in MacClade 4.08 [33]. Regions for which homology could not be confidently established were excluded from the phylogenetic analyses (see Table 1 and alignment in Supporting Information Dataset S1).

**Table 1:** Number of characters per gene that were excluded from and included in the phylogenetic analyses.

Gene	No. excluded characters	No. of retained characters	No. of parsimony informative characters	Best model AIC (weight)	Best model AICc (weight)	Best model BIC (weight)
16S	301	298	93			
COI	39	619	260			
Total	340	917	353	TVM+I+G (0.46)	TPM3uf+I+G (0.57)	TPM3uf+I+G (0.87)

The number of parsimony-informative characters is based on included characters only. Best model selected by jModeltest according to each criterion (AIC, AICc, BIC) and its corresponding weight.

For the phylogenetic analyses we also included sequences from all available species of *Ligia* in GenBank that had both genes and used as outgroup *Ligidum hypnorum*, a genus within the same family of *Ligia*, Ligiidae, to root the inferred phylogenetic trees. Ligiidae has only three genera [15]: *Ligia*, *Ligidium*, and *Ligidioides* (a monotypic genus from Australia). We used jModeltest v0.1.1 [34] to determine the most appropriate model of DNA substitution among 88 candidate models on a fixed BioNJ-JC tree, under the Akaike Information Criterion (AIC), corrected AIC(c), and Bayesian Information Criterion (BIC). For the ML analyses we used two different programs: (a) RaxML 7.0.4 [35]–[37] with two different models and number of bootstrap replicates determined automatically, as implemented in the CIPRES portal http://www.phylo.org/; and; (b) GARLI v.0.96beta8 [38] also implemented in CIPRES, with at least two different models and at least 100 bootstrap replicates. In addition, we conducted Bayesian analyses using the Parallel version of MrBayes v 3.1.2 [39], [40] implementing four runs, with four chains each, for at least 100,000,000 generations sampled every 5000 generations (all other parameters were default). Appropriate “burnin” (i.e., samples discarded prior to reaching a stationary posterior distribution) was determined based on small and stable average standard deviation of the split frequencies, Potential Scale Reduction Factor close to 1, and stable posterior probability values (see MrBayes manual).

For the Bayesian and RaxML analyses, we also conducted phylogenetic analyses with partitioned models in which each gene was in a different partition (i.e., each data partition assumed the same model but different parameter values).

**Figure 3 pone-0011633-g003:**
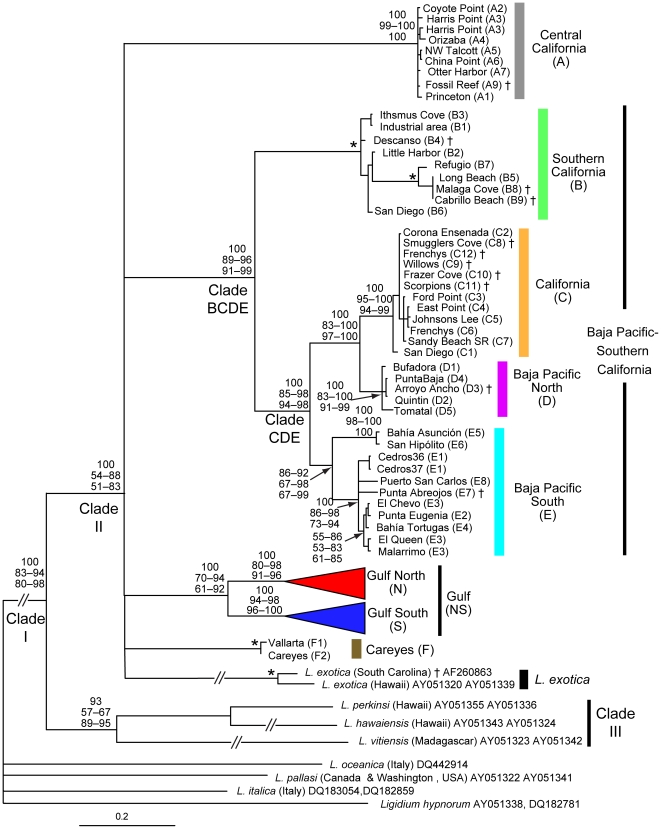
Maximum Likelihood tree of *Ligia* samples from localities in **Figure 1** and several outgroups. Obtained by RaxML for the 16S rDNA and COI genes (model GTR + G), rooted with *Ligidium*. Taxon IDs and clade colors correspond to Figure 2. *Ligia* Gulf clade portion of tree is expanded in Figure 4. Numbers by nodes indicate the corresponding range of node support values obtained for each method: Top-Bayesian Posterior Probabilities; Middle-GARLI bootstrap support; and Bottom-RaxML bootstrap support. * denotes nodes that received 100% support for all methods. Nodes receiving less than 50% support for all methods were collapsed. Nodes with no corresponding support values were of little relevance or had low support values. †: relationship based on 16S sequence only. San Diego (C1): GenBank Acc. Nos. AF260862 and AF255780.

To evaluate whether the *Ligia* sequences examined exhibit clocklike behavior, we used a Likelihood Ratio Test on the likelihood scores obtained with PAUP* [v. 4b10, 41] of the tree in Figure 3 with and without molecular clock constraints. Degrees of freedom were calculated as (number of taxa) –2 [42].

## Results

All new sequences have been deposited in GenBank under Acc. Nos. HM569779–HM570001 and our alignment has been deposited as Supporting Information Dataset S1. We removed 301 16S rDNA characters that could not be confidently aligned and the last 39 COI characters, which were missing for many taxa. The final dataset contained 917 characters of which 353 were parsimony informative (Table 1).

### Model selection

The best substitution model according to the AIC was TVM + G + I, while the best model according to AICc and BIC was TPM3uf + G + I (see jModeltest manual). These models were implemented in GARLI analyses, but they are not available in RaxML and MrBayes. In addition, the weights for these models were not high, particularly for the AIC and AICc (see Table 1) indicative of model selection uncertainty. The 99% cumulative weight interval included the most complex model evaluated (GTR + G + I). We therefore implemented the GTR + G + I, as well as the simpler GTR + G in RaxML, MrBayes, and GARLI, because of potential problems with estimating G + I simultaneously [see RaxML manual and pages 113–14 in 43].

### Phylogenetic results

Our phylogenetic analyses included all of the localities in Figure 2 as well as samples from all the *Ligia* spp. with sequences from both genes available in GenBank. *Ligidium hypnorum*, also in the family Ligiidae, was used to root our trees. Our results recovered a relatively well supported monophyletic group (Clade I; Fig. 3) that contained all of the *Ligia* sp. samples collected between central California and central Mexico, *L. exotica* samples from Hawaii and South Carolina, *L. perkinsi* (Hawaii), *L. hawaiensis* (Hawaii), and *L. vitiensis* (Madagascar), to the exclusion of samples of *L. oceanica* (Germany), *L. pallasi* (Canada and USA), and *L. italica* (Italy). Within Clade I, *L. perkinsi*, *L. hawaiensis*, and *L. vitiensis* appear as a sister clade (Clade III) to all the remaining samples (Clade II), although node support values for the reciprocal monophylies of Clades II and III were highly variable. Within Clade II, five monophyletic groups were recovered: central California clade (Clade A; grey in Figs. 2–3); Baja Pacific-Southern California clade (Clade BCDE; green, orange, magenta, and turquoise in Figs. 2–3); Gulf clade (Clade NS; red and blue in Figs. 1–3); Careyes clade (Clade F; brown in Figs. 2–3); and *L. exotica*. The relationships among the five clades within Clade II (i.e., A, BCDE, NS, F, and *L. exotica*) were unresolved. Relationships and phylogeographic patterns within each of these clades (except *L. exotica*) are described below.

The **central California clade** (Clade A; grey; Figs. 2–3) was comprised of mainland samples from the vicinity of the San Francisco Bay area and samples collected in the Northern Channel Islands. These island lineages are mainly located in the western portion of the Northern Channel Islands (west Santa Rosa and San Miguel), with the exception of Orizaba (A4) in Santa Cruz Island. Very shallow divergences were observed within this clade (maximum Kimura-2-parameter distances were 0.37% and 1.55%; for 16S rDNA and COI, respectively; Table S2).

The **Baja Pacific–Southern California** clade (i.e., BCDE) contained all the other samples collected from California and all the samples from the Baja Peninsula Pacific side. This clade was first divided into two groups: a group that contained samples from Santa Catalina Island and mainland southern California localities from San Diego to Refugio (“**Southern** **California”**; Clade B; green; Figs. 2–3); and a group that contained samples from the Baja Pacific, northern Channel Islands, and San Diego (i.e., Clade CDE). The CDE clade in turn was divided into two groups (i.e., E and CD). The first one (Clade E; “**Baja Pacific South**”; turquoise; Figs. 2–3) contained all of the Baja Peninsula Pacific localities south of Guerrero Negro Lagoon. The second one (i.e., CD; orange + magenta) contained all of the localities on the Baja Peninsula north of Guerrero Negro, a sample from San Diego, and several localities from the Northern Channel Islands. Clade CD was divided into a group that included only localities from the northern Pacific Baja Peninsula (Clade D; **Baja Pacific North**; magenta; Figs. 2–3), and a group that contained mainland localities from Ensenada (Mexico) and San Diego, and Northern Channel Islands localities (Clade C; **“California”**; orange; Figs. 2–3). These island localities are distributed in the eastern portion of the Northern Channel Islands (Anacapa, Santa Cruz, and eastern Santa Rosa).

Maximum sequence divergences within each of the B, C, D and E clades reached 2.22, 0, 0, 2.41%; respectively, for the 16S rDNA gene (Table S2), whereas they reached 8.60, 2.10, 2.08, 8.77%; respectively for the COI gene (Table S2, S3, S4, S5). Divergences among clades B, C, D and, E ranged between 1.02–7.5% for 16S rDNA and between 7.28–19.6% for COI (Table S2).

The **Gulf clade** (Clade NS; red and blue; Figs. 2–4); grouped all the localities that were sampled in the Gulf of California and all mainland samples collected south of the Gulf, with the exception of Vallarta and Careyes (brown in Figs. 2–3). The Gulf clade was divided into two well-supported main lineages: Gulf North clade (clade N; red; Figs. 2–4) and Gulf South clade (clade S; blue; Figs. 2–4). Divergences between the Gulf North and Gulf South clades ranged between 5.32–11.09% for 16S rDNA and between 15.16–26.47% for COI (Table S2). COI divergences among selected localities/lineages within the Gulf North and Gulf South clades are shown in Tables S6 and S7; respectively. The **Careyes clade** (Clade F; brown) included only the samples from Vallarta and Careyes.

**Figure 4:**
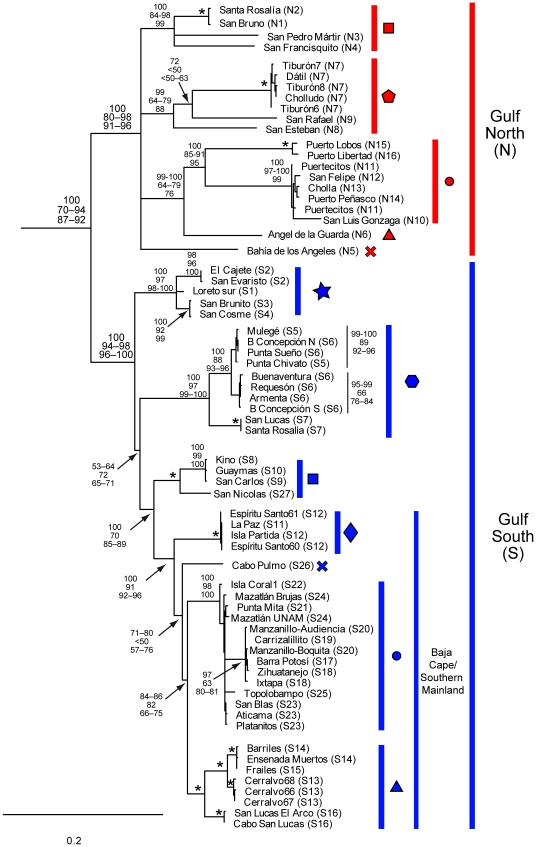
Maximum Likelihood tree of *Ligia* Gulf samples (expansion of the Gulf clade in **Figure 3**). Taxon IDs, clade colors and shapes correspond to localities in Figure 2. Numbers by nodes indicate the corresponding range of node support values obtained for each method: Top-Bayesian Posterior Probabilities; Middle-GARLI bootstrap support; and Bottom-RaxML bootstrap support. * denotes nodes that received 100% support for all methods. Nodes receiving less than 50% support for all methods were collapsed. Nodes with no corresponding support values were of little relevance or had low support values.

The **Gulf North** **clade** (Clade N; red) included: (1) Baja samples from San Bruno and Santa Rosalia (in the mid-peninsula), and all Baja localities sampled north of Santa Rosalia; (2) all Gulf mainland localities sampled from Puerto Libertad (N16) to the north; and (3) all samples collected from the central Gulf islands (Tiburón, Angel de la Guarda, San Esteban, San Pedro Martir, El Cholludo, Isla Datil; N3 and N7–N8). Within the Gulf North clade, a well-supported group (red circles; N10–N14; Figs. 2 and 4) was formed by localities from the upper Gulf between Puerto Peñasco (mainland), and San Luis Gonzaga (Baja), which was sister to a well-supported group formed by Puerto Lobos and Puerto Libertad (red circles; N15–N16). The sister lineage to this “red circles” clade (N10–N16) appears to be Angel de la Guardia Island (N6), but support for this relationship was variable. The island localities of Tiburon, El Cholludo and Isla Datil (N7) formed a well-supported group, whose closest relatives appear to be San Esteban Island (N8) and San Rafael (a Baja Peninsula locality; N9). San Francisquito, San Pedro Martir Island, Santa Rosalia and San Bruno formed a well-supported group (red squares N1–N4). The relationship of Bahía de los Angeles (N5) with the other lineages was not resolved.

The **Gulf South clade** (Clade S; blue) included: (1) San Lucas and Santa Rosalia (S7) and all populations south of San Bruno in the Baja Gulf; and all mainland populations collected from Kino (S9) to central Mexico (with the exception of Vallarta and Careyes). Baja Peninsula localities north of the Cape region (i.e., North of La Paz) were grouped into two well supported main clades: one (blue hexagons) included the localities sampled from Santa Rosalia and San Lucas to Bahía Concepción (S6), excluding San Bruno; the other (blue stars) included the localities from San Brunito to El Cajete. Baja Cape region lineages were grouped into three separate lineages: one that included all the localities from La Paz and Isla Espiritu Santo (blue diamonds); one that included all Baja Cape region localities south of La Paz with the exception of Cabo Pulmo (blue triangles); and one that included Cabo Pulmo (blue “X”; S26). These lineages formed a monophyletic group (i.e., “Baja Cape-Southern Mainland” clade; Fig. 4) with a clade comprised of all the mainland localities in the southern Gulf and those south of the Gulf (blue circles), with the exception of Vallarta and Careyes. The sister to the “Baja Cape-Southern Mainland” appears to be a clade (blue squares) formed by the central Gulf mainland localities of Kino, Guaymas, and San Carlos (S9–S10) and the central Baja locality of San Nicolas (S27).

## Discussion

### Taxonomic uncertainty and Central California Clade

Our inferred phylogenetic relationships among *Ligia* isopod samples from central California to central Mexico show a rather complex and previously unknown evolutionary history. The high degree of sequence divergence among several lineages suggests that multiple *Ligia* species exist. Recognized species of *Ligia* show COI divergences starting at 14% (*L. hawaiensis* vs. *L. perkinsi*; Table S2). Many of the lineages found in our study area are at least 14% divergent from their closest relative (Tables S2 and S6–S7), although smaller divergences may also represent different species. Accordingly, the taxonomy and systematics of *Ligia* in this region need to be revised. Traditionally, with the exception of *L. pallasi* in central California, one species has been recognized in this region, *Ligia occidentalis*; although some authors also indicate the presence of *L. baudiniana* (a species from the Atlantic) and *L. exotica* (a cosmopolitan species, but see discussion below) [15], [16]. The type locality of *Ligia occidentalis* Dana, 1853 is around the San Francisco area we sampled; thus, our samples from this region likely correspond to this species. These samples clustered into the **Central California Clade** (Clade A; grey), with several samples from the Northern Channel Islands. The small divergences observed among the localities within this clade (Table S2) suggest a recent exchange between the mainland and the islands. However, this clade is highly divergent from the other main lineages (Table S2), suggesting they correspond to different species. The regions between Point Conception and San Francisco and north of San Francisco need to be explored to determine the distributional limits of clades A, B, and C.

The four main lineages found in our study area (i.e., clades A, BCDE, Gulf, and Careyes) are closely related to *L. exotica*, *L. hawaiensis*, *L. perkinsi*, and *L. vitiensis*. In contrast, *L. pallasi*, a coastal species whose distribution overlaps with our study area (distributed between Santa Cruz, California, and Alaska), and for which we included samples from Canada and Washington, USA, is very distant from the *Ligia* examined in this study. *Ligia exotica* has been considered a single exotic species, commonly found in harbors and ports around the world, and thus regarded as introduced by ship traffic in many areas [reviewed in 15]. However, molecular analyses of samples from different parts of the world also show deep divergences among lineages, suggesting *L. exotica* is a complex of cryptic species (Hurtado et al., unpublished). Similarly, *L. hawaiensis* and *L. perkinsi*, both from the Hawaiian archipelago, show deep interpopulation genetic divergences, but the phylogenetic relationships among populations of the two members have not been well resolved [11]. To resolve the sister relationships of *Ligia* lineages found in our study area, a comprehensive examination that includes representatives of potential sister lineages and additional genes is necessary.

### Baja Pacific – Southern California (BCDE) Clade

Another deep lineage includes the clades B, C, D, and E. In the Southern California clade (Clade B; green), mainland localities from Los Angeles area (B5, B8, B9) to Refugio Beach (B7) formed a well-supported group. The localities sampled around the Los Angeles region (i.e., Long Beach, Malaga and Cabrillo) have identical 16S sequences suggesting recent exchange, which may be a consequence of the relatively young age of the current rocky headlands in southern California [1]. This group plus Refugio split from the rest of the members in the Southern California clade (B), which includes samples from San Diego and Santa Catalina Island, suggesting a vicariant event around Los Angeles region (represented by 6.89–8.04% COI divergence; Table S3). Remarkably, interspecific phylogeographic breaks have also been identified in the Los Angeles region for other intertidal and coastal animal taxa [44], implying they may share a common vicariant history in this region. The small divergences (0.9–2.42% COI; Table S3) observed between Santa Catalina Island localities (B1–B3) and the mainland (i.e., San Diego B6) may indicate a recent exchange, consistent with the young age of the island (∼1 Mya), which is suggested to have separated from southern California [45].

Within the California-Baja Pacific North-Baja Pacific South clade (i.e., CDE clade), a mid-peninsular phylogeographic break occurs at the Guerrero Negro Lagoon area (i.e., California + Baja Pacific North *vs*. Baja Pacific South). Thus, presence of this Lagoon may be associated with this phylogeographic split. Remarkably, a mid-peninsular phylogeographic break is also observed in *Ligia* lineages along the Baja peninsula inside the Gulf (i.e., Gulf North *vs.* Gulf South clades). A seaway may be the cause of the breaks on both sides of Baja (but see Gulf Clade Discussion).

Divergence of the Baja Pacific North Clade (D; magenta) from the California Clade “C” (orange) may be associated with one of the multiple marine incursions that are suggested to have isolated the peninsula from mainland between 5–14 Ma [reviewed in 46]. Within the California Clade C (orange) the small COI divergences observed (maximum 2.1%; Tables S2 and S4) among the northern Channel Island localities and between these and their closest relatives on the mainland (i.e., C1 and C2), suggest recent exchange among these localities (similar to the Channel island populations of the Central California clade A; grey). These islands appear to have formed ∼3 Ma [N. Pinter pers. comm. in 47]. However, Anacapa and San Miguel islands were completely under water during at least one interglacial episode [45], so *Ligia* populations present before would have gone extinct. Santa Rosa and Santa Cruz islands have been continuously above sea level for at least the last 500,000 years [45]; but it is possible that they were inundated before. The four present-day Northern Channel Islands were all part of a large contiguous land mass ∼17,000 years ago, which is believed to have been connected to the mainland and facilitated dispersal of terrestrial animals to this island [45]. Based on the distribution of non-vagile reptiles, it has been also suggested that the Northern Channel Islands were once connected to mainland, near present-day Mexico, and were carried farther north along fault systems [45]. The grouping of these Northern Channel Islands lineages with samples from San Diego and Ensenada suggests an origin of the island lineages around the Mexico-US border. Interestingly, the distribution of the two main lineages found in the Northern Channel Islands is segregated geographically: California clade C lineages (orange) in the east; and Central California clade lineages (grey) in the west, which may be related to the past history of colonization and fragmentation of the islands.

Experimental reciprocal crosses provide further evidence for multiple species of *Ligia* in California [16]. Crosses between individuals from Los Angeles and Carpenteria (found between B7 and B8; Fig. 2) produced normal offspring. However, no offspring were observed in crosses of Carpenteria and San Francisco, which were found to be highly divergent in our study; as well as between Carpenteria and the Channel Islands of San Nicolas and San Clemente, which were not sampled in our study.

### Gulf Clade

The Gulf clade is characterized by extremely high genetic divergences among and within several lineages (north vs. south COI divergences: 15.16–26.47%; within north: up to 25.3%; and within south: up to 21.55%; Supporting Tables S2 and S6–S7), suggestive of a long history in the region. Due to the high allopatric divergences among the lineages within this clade, its apparently long history in the region, and a broad geographic distribution in the Gulf and adjacent areas, *Ligia* has the potential to have retained in its phylogeographic patterns, signatures of past vicariant events in this region. However, interpretation of this pattern is limited by the incomplete and controversial state of knowledge on the complex geological history of the Gulf of California and Baja California Peninsula; for which alternative and strikingly different hypotheses have been proposed [e.g. 21],[22]–[27].

The mid-peninsular breaks observed for *Ligia* at the Pacific (i.e., Guerrero Negro area) and Gulf (i.e., Santa Rosalia area) sides of Baja appear to be consistent with the presence of a seaway, because such a barrier would likely cause north-south breaks at both sides of the peninsula for a coastal organism. However, the mid-peninsular break in the Pacific is shallower than the one observed in the Gulf, although substitution rate heterogeneity, which is observed in our dataset (*P*<2.05×10^−17^; *d.f.* = 130), could account for the different divergences. Although the break around Santa Rosalia is consistent with the proposed location for a seaway opening, the break in the Pacific is ∼170 Km north of the suggested location (black circle; Fig. 2) for a seaway opening in the Pacific [48]. Therefore, it is unclear whether or not the Pacific and Gulf mid-peninsular breaks are associated with the same event.

Existence of a mid-peninsular seaway has been a contentious issue in phylogeographic and geological studies. A ∼1 Ma mid-peninsular seaway was originally proposed to explain a mid-peninsular divergence of *Uta* lizards [28], but the same research group indicates in subsequent papers that this divergence may represent Late Miocene–Early Pliocene times [24], [49], [50]. Mid-peninsular phylogeographic breaks have been reported in multiple taxa [e.g., 22]; but a broad range of divergences is observed, with some showing deep [46], [49], [51], while others shallower phylogeographic breaks [22]. Riginos [52] suggests that a Plio-Pleistocene mid-peninsular seaway is the simplest explanation for a concordant genetic division within both terrestrial and marine vertebrates. However, a comparative phylogeographic analysis using data from multiple studies concludes that two mid-peninsular diversification events occurred [53]. In addition, Grismer [54] indicates that mid-peninsular divergences may be associated with abrupt habitat and climate changes in central Baja, rather than a seaway. No geological evidence for a mid-peninsular seaway ∼1 Ma exists [24], but geological evidence (albeit controversial) suggests the existence of a more ancient seaway. Based on characteristics and distributions of fossil assemblages and marine deposits, Helenes and Carreño [26] suggest a Miocene seaway connected the Pacific with a northern proto-Gulf basin (discussed below) through the central part of Baja. However, Oskin and Stock [55] consider that a seaway does not necessarily explain the distribution of Miocene marine deposits in Baja. Nevertheless, paleomagnetic data indicate the presence of a seaway in Santa Rosalia ∼7 Ma [56].

Regardless of whether or not a seaway was the cause for the Gulf mid-peninsular break in *Ligia*, it does not appear to be a ∼1 Ma event. The high divergence between Gulf North and Gulf South clades (COI Mean  = 21.68%±1.9 SD) suggests a divergence time older than 1 Ma, probably in the Miocene, unless very elevated substitution rates occur in *Ligia*, which is unlikely. The mutation rate of COI in other marine isopods is suggested to be 2.5%/My [57]; thus, for this divergence to represent 1 My, it implies *Ligia* has a substitution rate ∼9 times higher than that reported for other marine isopods.

Other aspects of the phylogeographic patterns of *Ligia* are incongruent with the hypothesis of Riddle [i.e., Fig. 1; 22]. Under Riddle's hypothesis, reciprocal monophylies of the mainland *vs*. the peninsula (or at least the monophyly of one of them) are expected. However, within the Gulf, *Ligia* lineages of southern Baja are more closely related to mainland lineages (south of ∼29°N latitude) than to northern Baja and northern mainland lineages. Furthermore, *Ligia* Cape region lineages are more closely related to southern mainland lineages (south of ∼25°N latitude) than to any other Baja lineages. This is in striking contrast to the suggestion that the Baja Cape region was the first part of the peninsula to separate from mainland [22], [55]. Dispersal of *Ligia* across long stretches of ocean between southern Baja and mainland may explain these discrepancies. We believe this is unlikely because if *Ligia* from the Gulf of California had such dispersal abilities (i.e., peninsula–mainland distances are >100 Km in the central Gulf and >190 Km in the mouth), we would not expect to see the exceptional degree of allopatric differentiation exhibited. Furthermore, mixing of distant localities by dispersal across long stretches of ocean would likely have produced a very random phylogeographic pattern, which, in general, does not appear to be the case. Nevertheless, such dispersal cannot be completely ruled out. However, the phylogeographic patterns of *Ligia* in the Gulf clade appear to be congruent with elements of alternative geological hypotheses that consider an older history for the Gulf, as well as with phylo- and biogeographic patterns of other taxa.

The existence of an isolated Late-Miocene proto-Gulf basin that included the northern portion of today's Gulf and an extensive area to the north is well-accepted and was proposed since the 1970's [25], [58]–[61]. Recent evidence suggests that this northern proto-Gulf is at least 11.61 Ma old [60]. As mentioned above, a connection between the proto-Gulf and the Pacific appears to have existed in the central part of the peninsula [25], [26], [48]. How the Gulf evolution proceeded from the northern proto-Gulf stage to the present-day Gulf is unclear [25]. Nevertheless, several studies suggest that the southern part of Baja separated from mainland more recently than northern Baja, and that the Cape region was the last part of Baja to separate from mainland ∼4–6 Ma [25], [26], [61]. Such sequence of events is consistent with the phylogeographic patterns of *Ligia* in the Gulf South clade. Ledesma-Vásquez [62] proposes that a southern basin formed, younger (3.5–5.5 Ma) than the northern proto-Gulf, and that the older northern proto-Gulf joined this southern basin to form the present-day Gulf; although the exact sequence of events is unclear. Accordingly, the present-day Gulf attained its current form as a result of the separation of the Cape region from mainland and the break of the land barrier separating the two basins. Therefore, some present-day Gulf taxa, including *Ligia*, may have colonized and remained in the Gulf since northern proto-Gulf times. An early diversification (i.e., during proto-Gulf rather than modern Gulf times) may have contributed to the high levels of endemism observed in the northern Gulf [19].

Phylogeographic patterns of other taxa are also suggestive of colonization and isolation events during proto-Gulf times. Several fish lineages with disjunct distributions in the Pacific coast of Baja and the northern Gulf (i.e., not found in southern Gulf) show relatively deep Pacific-northern Gulf divergences. For example, divergence between *Gillichthys mirabilis* (with disjunct distribution) and its sister *Gillichthys seta*, endemic to the northern Gulf, is estimated to have occurred 4.6–11.6 Ma [63]. Similarly, Pacific/Gulf divergences for *Leuresthes tenuis*, *Girella nigricans*, and *Hypsoblennius jenkins* (other fish taxa with disjunct distributions) at the mitochondrial control region are 11.6%, 8.5%, and 7.9%, respectively [64]. According to a molecular clock of 0.85–2% per million years presumably specific to fish mitochondrial control region [52], some of the deeper divergences may have occurred during Miocene times (i.e., proto-Gulf times). Nevertheless, much shallower Pacific/northern Gulf control region divergences are reported for other fish taxa [0.6–2.3%, 64] with disjunct distributions, suggesting that more recent colonization events of the northern Gulf have also occurred.

As discussed above, our results suggest that *Ligia* colonized the Gulf during proto-Gulf times; and that the Cape region was the last portion of southern Baja to separate from the southern mainland. Interpretation of the sequence of events that led to the divergence of the *Ligia* Gulf clade from its sister lineage in the Pacific (yet to be identified or extinct), and to the subsequent divergence of the Gulf North and Gulf South clades is limited by the incomplete knowledge of the geological history. Below, we identify three possible vicariant scenarios that could explain the early history of the *Ligia* Gulf clade, although geological evidence for several aspects of these scenarios does not exist.

#### Scenario 1

The ancestor of the Gulf clade colonized the proto-Gulf via a Pacific-proto-Gulf connection that was later disrupted, causing the divergence of the Gulf clade from its sister lineage. Subsequently, one of the following two scenarios occurred. **Scenario 1A.** A mid-peninsular seaway splits the Gulf clade into north and south. Subsequently the Gulf North clade disperses along the coast in the northern part of the Gulf, while the Gulf South clade disperses along the southern coast and through the existing land bridge between the southern peninsula and the mainland. The two lineages do not disperse beyond their present-day limits in the mainland (around Tiburon Island) because rocky habitats are already occupied by the other lineage. **Scenario 1B.** Formation of a land bridge divides the proto-Gulf into two separate basins (north and south), each with its respective southern end closed, causing divergence of the Gulf North and Gulf South Clades.

#### Scenario 2

The ancestor of the Gulf clade diverged from its sister in the Pacific prior to entering the proto-Gulf. Subsequently, the ancestor of the Gulf North clade (rather than the ancestor of the whole Gulf Clade as in Scenario 1) colonizes the northern proto-Gulf, while the ancestor of Gulf South Clade remains outside the proto-Gulf in the Pacific or in a separate southern basin.

The exact sequence of events following divergence of the Gulf North and Gulf South clades is also unclear, but several patterns are worth noting. First, if the Gulf North clade indeed diverged from the Gulf South clade in a separate northern basin, presence of the Gulf North clade in the central Gulf (“Midriff”) islands sampled, may indicate that these islands formed part of the southern end of the northern proto-Gulf basin. If Tiburon Island formed part of the eastern end of the land bridge that separated the two basins, this would explain the limits of the Gulf North and Gulf South clades in the mainland (i.e., Puerto Libertad (N16) and Kino (S9); respectively). Second, within the Gulf South clade, the sister relationship between San Nicolas (S27; Figs. 2 and 4) in the peninsula and the Guaymas-Kino-San Carlos clade (S9–10) in the mainland is consistent with the suggestion of Ledezma-Vásquez et al. [65] that San Nicolas was attached to the central Gulf mainland through the land bridge that constituted the northern limit of the southern basin ∼3.3. Ma [62]. Third, deep divergences among clades of geographically close localities in southern Baja (i.e., blue hexagons, starts, diamonds, and triangles: Figs. 1 and 3), suggests a long-standing isolation of these regions. A deep phylogeographic break in the region north of Loreto such as the one observed in *Ligia* (i.e., blue stars vs. hexagons) is reported for a lizard [49]. Fourth, Santa Rosalia is the only locality where members of both, the Gulf North and Gulf South clades, were found. However, the haplotypes are identical to the ones found in the nearest localities, San Lucas (Gulf South) and San Bruno (Gulf North), suggesting a recent colonization of Santa Rosalia. Finally, shallow divergences across extensive distances indicate recent colonization/fragmentation processes in *Ligia* Gulf populations. For example, it is not surprising that the Upper Gulf region (red circles; Figs. 1 and 3) exhibits little divergence among localities, since this is the shallowest part of the Gulf and therefore likely to have been significantly contracted during the low sea level periods of the Pleistocene.

*Xantusia* lizards show similar phylogeographic patterns to the *Ligia* Gulf Clade. These lizards share several features with *Ligia*. They have a circum-Gulf of California distribution; comprise a large number of allopatric populations with high levels of genetic divergence; and, have an extremely patchy distribution, limited vagility, and close association with particular structural niches [51]. As in *Ligia*, northern and southern Baja lineages of *Xantusia* cluster in two separate clades that are highly divergent (i.e., ∼22% for *Cytb* and ND4 mitochondrial genes); possibly representing a Miocene separation [51]. In addition, *Xantusia* lineages from southern Baja, including the Cape region, are more closely related to lineages in mainland localities that are found inland south of latitude 25°N (see Fig. 2); and, as in *Ligia*, their divergence occurred more recently than the mid-peninsular separation. Across-ocean dispersal is very unlikely to explain this relationship in *Xantusia*, because the southern mainland lineages are found far inland from the coast.

Other extant and fossil taxa show also a close association between the Baja Cape region and the southern mainland. These include many reptilian taxa [23], [24], the rocky intertidal snail *Tegula ligulata* [66], the lichen *Phloeopeccania anemoides* [67], and fossil vertebrates [68].

Many terrestrial vertebrates, however, show a monophyly of the peninsula with respect to mainland [22], [53], [69], instead of the patterns observed in *Ligia* (i.e., monophyly of southern Baja and mainland localities south of ∼29°N latitude; and monophyly of the Cape region with southern mainland localities south of ∼25°N latitude). If these patterns in *Ligia* are indeed the result of vicariance, why don't these other taxa show evidence of such vicariant events? First, it is possible that other taxa colonized the southern portion of the peninsula from the north after it had separated from the mainland and had become part of the rest of the peninsula, rather than prior to the formation of the Gulf's southern mouth. Second, most studies do not have the necessary geographic sampling to test whether the Cape Region is most closely related to the southern mainland [e.g. 46], [49], [70], because they lack samples from the southern mainland (i.e., the mainland region to which the southernmost portion of the peninsula was presumably attached). This is particularly problematic for terrestrial taxa restricted to the desert, because the southern mainland counterpart's habitat is tropical deciduous forest, thus, outside their present distribution range. *Ligia*, on the other hand, is unlikely to be affected by inland habitat changes as suggested by its wide distribution. Perhaps the only phylogeographic studies that do include samples from the southern mainland are on *Xantusia* lizards [51] and on *Trimorphodon* snakes [69]. As mentioned above, *Xantusia* shows a similar pattern to *Ligia*. In contrast, *Trimorphodon* exhibits a monophyly of the whole peninsula (including southern California), which is more consistent with the traditional vicariant scenario (i.e., south to north opening of the Gulf of California).

Our interpretation of the phylogeographic patterns of *Ligia* would likely benefit from divergence time estimations with relaxed clock methodologies. Unfortunately, no reliable calibration points are available for the phylogeny of *Ligia*, such as dated fossils or vicariant events. Furthermore, application of substitution rates from other taxa is not appropriate because the assumption of molecular clock in our data is violated. Although we identify nodes in the phylogeny that could be attributed to dated vicariant events (e.g., the Cape region vs. mainland separation, mid-peninsular divergence(s), and the San Nicolas vs. Guaymas-San Carlos-Kino divergence), these nodes are also among the most controversial ones regarding their actual timing and/or whether or not they represent dispersal rather than vicariance in *Ligia*. Thus, calibrating a clock at these nodes would lead to questionable conclusions.

### Careyes Clade

This clade, which represents another highly divergent lineage in our study area, is enigmatic because of its limited and peculiar distribution. It was found in only two localities (Puerto Vallarta and Careyes) separated by ∼94 Km of coastline; spanning a region that interrupts the distribution of one of the Gulf South clades (blue circles; Figs. 2 and 4). Gulf South lineages found south of the Careyes clade, form a monophyletic group (i.e., Manzanillo S20 to Barra Potosi S17) that represents a much younger lineage than the Careyes clade. How this Gulf clade ‘skipped’ the region occupied by the Careyes clade is intriguing. The divergence of the Careyes clade from the other lineages in our study may be related to the suggested formation of a Miocene (∼8 Ma) microbasin around Puerto Vallarta [46].

### Concluding remarks, implications, and future work

As expected from its biology, extraordinary levels of allopatric genetic divergence among many localities of *Ligia* were observed in our study area. The spatial distribution of well-supported *Ligia* monophyletic groups corresponds strongly with geography, with little spatial overlap among clades; suggesting allopatric differentiation plays a major role in diversification of *Ligia* in this region. Extremely high mitochondrial genetic divergence is observed among the four main *Ligia* lineages identified, and within both, the Baja Pacific-Southern California and the Gulf clades. Although *L. occidentalis* is the only species usually recognized in the study area (in addition to *L. pallasi* in central California), high levels of genetic differentiation suggest the existence of multiple species. Indeed, based on our results and the type locality, only the Central California Clade would correspond to *L. occidentalis*. Therefore, the taxonomy of *Ligia* in the study area needs to be revised. This is important for conservation, as some divergent lineages have a very restricted distribution and, thus, are vulnerable to local anthropological pressures on the rocky intertidal.

Relationships among the four main lineages found in our study area could not be resolved. However, they are part of a well-supported clade that contains *L. exotica*, *L. perkinsi*, *L. hawaiensis*, and *L. vitiensis*; and excludes *L. pallasi* (distributed from central California to Alaska), as well as all other *Ligia* species examined. Inclusion of additional markers and *Ligia* lineages may help resolve these relationships.

Differentiation of many divergent regional clades of *Ligia* in our study area may be associated with past vicariant events. In contrast, shallow divergences within some of these clades across extensive distances may indicate recent exchange. For example, recent low sea levels may have facilitated recent exchange among localities in the upper Gulf and among localities within the mainland area south of the Gulf.

The phylogeographic patterns of *Ligia* in the Gulf of California and Baja Peninsula deviate from the pattern expected under a vicariant scenario often used by phylogeographers. This is surprising given that *Ligia* has characteristics of a taxon with a high potential for retaining phylogeographic signatures of past vicariant events that occurred in the study region (i.e., wide distribution, high allopatric differentiation, very divergent lineages, and extremely limited dispersal abilities). Although long-distance over-water dispersal cannot be completely ruled out as the cause of these conflicting patterns, the life history, exceptional degree of allopatric divergences, and the non-random phylogeographic pattern of *Ligia*, argue against this kind of dispersal as the cause of this discordance. Furthermore, some of the most “conflicting” phylogeographic patterns of *Ligia* appear to be concordant with aspects of alternative geological hypotheses that encompass an older origin of the Gulf. In this regard, it is surprising that the phylogeographic literature of this region conveys an impression that the geological history is well resolved and agreed upon, while this issue is subject to intense and ongoing debate among geologists. Congruence between the phylogeographic patterns of an organism with the characteristics of *Ligia* and an alternative geological hypothesis will contribute to this debate, and underscores that alternative hypotheses should be considered when examining phylogeographic patterns in this region. Examination of other taxa with restricted dispersal abilities, whose ancestors inhabited the region prior the formation of the Gulf of California, and whose current distribution includes key areas such as the southern Baja peninsula and the southern mainland, will allow for an adequate examination of alternative geological hypotheses. Shared patterns among multiple taxa with these characteristics should shed light on the complex history of this important biogeographic region. In addition, nuclear markers of *Ligia* should be examined to corroborate that the mitochondrial phylogeographic patterns extend to the rest of the genome.

## Supporting Information

Dataset S1 Alignment of the 16S ribosomal DNA gene and Cytochrome C Oxidase Subunit I (COI) gene sequences used in this study in Nexus format.
(0.18 MB TXT)

Table S1 Sampled localities and corresponding latitude and longitude (where available). IDs correspond to labels used in Figures.
(0.14 MB DOC)

Table S2 Ranges of Kimura-2-parameter distances observed among main *Ligia* clades found in our study area and outgroups. Upper matrix: COI gene distances. Lower matrix: 16S rDNA gene distances. Values on diagonal show maximum within-clade divergence (left: COI gene; right: 16S rDNA gene).
(0.07 MB DOC)

Table S3 Cytochrome Oxidase I (COI) gene percent divergence (Kimura-2-parameter correction) ranges within (diagonal) and among (below diagonal) selected lineages in the Southern California clade (B; green in Fig. 3).
(0.04 MB DOC)

Table S4 Cytochrome Oxidase I (COI) gene percent divergence (Kimura-2-parameter correction) among localities in the California 2 clade (C; orange in Fig. 3).
(0.03 MB DOC)

Table S5 Cytochrome Oxidase I (COI) gene percent divergence (Kimura-2-parameter correction) ranges within (diagonal) and among (below diagonal) selected groups of localities in the Baja Pacific South clade (BPS; turquoise in Fig. 3).
(0.05 MB DOCX)

Table S6 Cytochrome Oxidase I (COI) gene percent divergence (Kimura-2-parameter correction) ranges within (diagonal) and among (below diagonal) selected groups of localities in the Gulf North clade (red in Figs. 3 and 4). Shapes refer to clades defined in Fig. 4.
(0.05 MB DOC)

Table S7 Cytochrome Oxidase I (COI) gene percent divergence (Kimura-2-parameter correction) ranges within (diagonal) and among (below diagonal) selected groups of localities in the Gulf South clade (blue in Figure 4). Shapes refer to clades defined in Fig. 4.
(0.08 MB DOC)
