# Supplementary material for: Phylogeography of Supralittoral Rocky Intertidal Ligia Isopods in the Pacific Region from Central California to Central Mexico
Source: PLoS One. 2010 Jul 21;5(7):e11633. doi: 10.1371/journal.pone.0011633 (PMC2908127; doi:10.1371/journal.pone.0011633)
Supplement: Table S1 — Sampled localities and corresponding latitude and longitude (where available). IDs correspond to labels used in Figures. (0.14 MB DOC) [file pone.0011633.s002.doc]

| **Locality** | **ID** | **Latitude** | **Longitude** |
| --- | --- | --- | --- |
| Princeton, San Francisco Bay, California, USA | A1 |  |  |
| Coyote Point, San Francisco Bay, California, USA | A2 |  |  |
| Harris Point, San Miguel Island, California, USA | A3 | 34° 4'24.12"N | 120°21'47.58"W |
| Orizaba, Santa Cruz Island, California, USA | A4 | 34° 2'42.30"N | 119°43'18.78"W |
| NW-Talcott, Santa Rosa Island, California, USA | A5 | 34° 0'34.44"N | 120°13'3.36"W |
| China Point, Santa Rosa Island, California, USA | A6 |  |  |
| Otter Harbor, San Miguel Island, California, USA | A7 | 34° 3'7.32"N | 120°24'27.30"W |
| Fossil Reef, Santa Rosa Island, California, USA | A8 | 33°59'35.34"N | 120°14'15.30"W |
| Industrial area, Catalina Island, California, USA | B1 |  |  |
| Little Harbor, Catalina Island, California, USA | B2 |  |  |
| Ithsmus Cove, Catalina Island, California, USA | B3 |  |  |
| Descanso, Catalina Island, California, USA | B4 |  |  |
| Long Beach, California, USA | B5 |  |  |
| San Diego, California, USA | B6 |  |  |
| Refugio, California, USA | B7 |  |  |
| Malaga Cove, California, USA | B8 |  |  |
| Cabrillo, California, USA | B9 |  |  |
| San Diego, California, USA | C1 |  |  |
| Corona Beach, Ensenada, Baja California, Mexico | C2 | 31°47'23.40"N | 116°36'57.36"W |
| Ford Point, Santa Rosa Island, California, USA | C3 | 33°54'52.68"N | 120° 3'2.46"W |
| East Point, Santa Rosa Island, California, USA | C4 | 33°56'33.72"N | 119°58'4.68"W |
| Johnsons Lee, Santa Rosa Island, California, USA | C5 | 33°54'31.62"N | 120° 5'12.48"W |
| Side Frenchy’s, Anacapa Island, California, USA | C6 |  |  |
| Sandy Beach, Santa Rosa Island, California, USA | C7 |  |  |
| Smugglers Cove, Santa Cruz Island, California, USA | C8 | 34° 1'10.20"N | 119°32'19.20"W |
| Willows Anchorage, Santa Cruz Island, California, USA | C9 | 33°57'42.60"N | 119°45'17.82"W |
| Fraser Cove, Santa Cruz Island, California, USA | C10 | 34° 3'45.54"N | 119°55'8.94"W |
| Scorpion Pier, Santa Cruz Island, California, USA | C11 | 34° 2'43.86"N | 119°32'48.66"W |
| Frenchy’s, Anacapa Island, California, USA | C12 | 34° 0'23.76"N | 119°24'38.76"W |
| Bufadora, Ensenada, Baja California, Mexico | D1 | 31°43'28.62"N | 116°43'12.30"W |
| Bahía San Quintin, Baja California, Mexico | D2 | 30°18'35.03"N | 115°49'0.47"W |
| Punta Baja, Baja California, Mexico | D3 | 29°57'18.01"N | 115°48'26.08"W |
| Arroyo Ancho, Baja California, Mexico | D4 | 29°54'44.88"N | 115°42'40.50"W |
| Tomatal, Baja California, Mexico | D5 | 28°29'12.53"N | 114° 4'5.04"W |
| Isla Cedros, Baja California, Mexico | E1 | 28° 6'2.22"N | 115°11'10.98"W |
| Isla Cedros, Baja California, Mexico | E1 | 28° 5'28.44"N | 115°17'7.80"W |
| Isla Cedros, Baja California, Mexico | E1 | 28° 4'54.48"N | 115°20'19.68"W |
| Isla Cedros, Baja California, Mexico | E1 | 28° 7'8.22"N | 115°20'34.80"W |
| Punta Eugenia Isla Cedros, Baja California Sur, Mexico | E2 | 27°51'6.96"N | 115° 4'39.41"W |
| El Chevo, Baja California Sur, Mexico | E3 | 27°48'34.55"N | 114°50'13.22"W |
| El Queen, Baja California Sur, Mexico | E3 | 27°46'39.48"N | 114°38'22.69"W |
| Malarrimo, Baja California Sur, Mexico | E3 | 27°48'19.32"N | 114°43'32.82"W |
| Bahía Tortugas, Baja California Sur, Mexico | E4 | 27°41'29.28"N | 114°53'30.18"W |
| Bahía Asunción, Baja California Sur, Mexico | E5 | 27° 8'12.78"N | 114°17'43.56"W |
| San Hipolito, Baja California Sur, Mexico | E6 | 26°59'26.76"N | 113°58'38.94"W |
| Punta Abreojos, Baja California Sur, Mexico | E7 | 26°46'55.92"N | 113°30'50.53"W |
| Puerto San Carlos, Baja California Sur, Mexico | E8 | 24°47'51.78"N | 112° 6'57.90"W |
| Vallarta, Jalisco, Mexico | F1 | 20°36'55.62"N | 105°13'58.86"W |
| Careyes, Jalisco, Mexico | F2 | 19°26'13.50"N | 105° 1'38.94"W |
| Loreto, Baja California Sur, Mexico | S1 | 25°52'43.50"N | 111°20'31.56"W |
| Cajete, Baja California Sur, Mexico | S2 | 24°15'21.06"N | 110°36'41.82"W |
| San Evaristo, Baja California Sur, Mexico | S2 | 24°21'4.32"N | 110°40'37.38"W |
| San Brunito, Baja California Sur, Mexico | S3 | 26°12'59.40"N | 111°22'40.62"W |
| San Cosme, Baja California Sur, Mexico | S4 | 25°34'40.74"N | 111°10'0.00"W |
| Punta Chivato, Baja California Sur, Mexico | S5 | 27° 3'53.94"N | 111°57'28.68"W |
| Mulegé, Baja California Sur, Mexico | S5 | 26°54'7.56"N | 111°57'14.46"W |
| Bahía Concepción N, Baja California Sur, Mexico | S6 | 26°49'46.32"N | 111°52'20.64"W |
| Punta Sueño, Bahía Concepción, Baja California Sur, Mexico | S6 | 26°47'56.82"N | 111°51'48.72"W |
| Buenaventura, Bahía Concepción, Baja California Sur, Mexico | S6 | 26°38'38.88"N | 111°50'46.68"W |
| Requesón, Bahía Concepción, Baja California Sur, Mexico | S6 | 26°38'22.68"N | 111°49'45.18"W |
| Bahía Armenta, Bahía Concepción, Baja California Sur, Mexico | S6 | 26°37'37.44"N | 111°48'52.26"W |
| Mirador, Bahía Concepción, Baja California Sur, Mexico | S6 | 26°35'42.24"N | 111°47'33.72"W |
| Monumento, Bahía Concepción, Baja California Sur, Mexico | S6 | 26°34'0.60"N | 111°46'52.92"W |
| San Lucas, Baja California Sur, Mexico | S7 | 27°13'31.62"N | 112°12'33.00"W |
| Santa Rosalía, Baja California Sur, Mexico | S7 | 27°21'40.62"N | 112°16'47.82"W |
| Kino, Sonora, Mexico | S8 | 28°51'54.06"N | 112° 1'54.30"W |
| San Carlos, Sonora, Mexico | S9 | 27°56'21.42"N | 111° 5'19.26"W |
| Guaymas, Sonora, Mexico | S10 | 27°54'44.33"N | 110°56'49.56"W |
| La Paz, Baja California Sur, Mexico | S11 | 24°13'45.60"N | 110°18'33.54"W |
| La Paz, Baja California Sur, Mexico | S11 | 24°12'0.90"N | 110°17'58.68"W |
| La Paz, Baja California Sur, Mexico | S11 | 24°10'48.36"N | 110°18'10.44"W |
| Isla Espiritu Santo, Baja California Sur, Mexico | S12 | 24°24'13.08"N | 110°20'53.28"W |
| Isla Espiritu Santo, Baja California Sur, Mexico | S12 | 24°26'25.80"N | 110°22'19.92"W |
| Isla Partida, Baja California Sur, Mexico | S12 | 24°31'56.58"N | 110°23'0.00"W |
| Isla Cerralvo, Baja California Sur, Mexico | S13 | 24° 8'33.72"N | 109°49'19.02"W |
| Isla Cerralvo, Baja California Sur, Mexico | S13 | 24° 9'19.92"N | 109°47'57.18"W |
| Isla Cerralvo, Baja California Sur, Mexico | S13 | 24° 8'50.94"N | 109°51'36.42"W |
| Ensenada de los Muertos, Baja California Sur, Mexico | S14 | 23°59'26.88"N | 109°49'29.10"W |
| Barriles, Baja California Sur, Mexico | S14 | 23°44'4.14"N | 109°42'46.92"W |
| Frailes, Baja California Sur, Mexico | S15 | 23°22'58.98"N | 109°25'21.48"W |
| Cabo San Lucas, Baja California Sur, Mexico | S16 | 22°53'53.46"N | 109°52'32.28"W |
| Cabo San Lucas, Baja California Sur, Mexico | S16 | 22°52'38.70"N | 109°53'51.64"W |
| Barra Potosi, Guerrero, Mexico | S17 | 17°32'12.30"N | 101°26'42.78"W |
| Ixtapa, Guerrero, Mexico | S18 | 17°40'55.14"N | 101°38'45.60"W |
| Zihuatanejo, Guerrero, Mexico | S18 | 17°37'29.22"N | 101°32'44.40"W |
| Playa Carrizalillito, Michoacán, Mexico | S19 | 18° 3'29.94"N | 102°42'14.40"W |
| Playa audiencia, Manzanillo, Colima, Mexico | S20 | 19° 6'19.68"N | 104°21'6.24"W |
| Boquita, Manzanillo, Colima, Mexico | S20 | 19° 6'10.74"N | 104°23'48.06"W |
| Punta Mita, Nayarit, Mexico | S21 | 20°46'16.62"N | 105°31'4.50"W |
| Isla Coral, Nayarit, Mexico | S22 | 21° 2'51.66"N | 105°16'16.08"W |
| San Blas, Nayarit, Mexico | S23 | 21°32'25.74"N | 105°17'29.52"W |
| Platanitos, Nayarit, Mexico | S23 | 21°21'11.40"N | 105°14'22.20"W |
| Aticama, Nayarit, Mexico | S23 | 21°29'8.40"N | 105°11'55.62"W |
| UNAM, Mazatlán, Sinaloa, Mexico | S24 | 23°11'1.98"N | 106°25'34.68"W |
| Playa Brujas, Mazatlán, Sinaloa, Mexico | S24 | 23°18'26.40"N | 106°29'21.66"W |
| Topolobampo, Sinaloa, Mexico | S25 | 25°36'9.91"N | 109° 2'19.09"W |
| Cabo Pulmo, Baja California Sur, Mexico | S26 | 23°25'47.46"N | 109°25'42.48"W |
| San Nicolas, Baja California Sur, Mexico | S27 | 26°32'37.02"N | 111°32'24.08"W |
| San Bruno, Baja California Sur, Mexico | N1 | 27° 9'46.26"N | 112° 9'34.14"W |
| South of San Bruno, Baja California Sur, Mexico | N1 | 27° 9'28.32"N | 112° 7'56.58"W |
| Santa Rosalía, Baja California Sur, Mexico | N2 | 27°21'40.62"N | 112°16'47.82"W |
| Isla San Pedro Martir, Sonora, Mexico | N3 | 28°22'47.16"N | 112°19'7.74"W |
| San Francisquito, Baja California, Mexico | N4 | 28°25'17.58"N | 112°50'55.98"W |
| Bahía de los Angeles, Baja California, Mexico | N5 | 28°54'19.62"N | 113°32'2.40"W |
| Las Gringas, Bahía de los Angeles, Baja California, Mexico | N5 | 29° 1'20.82"N | 113°33'38.94"W |
| Isla Angel de la Guarda, Baja California, Mexico | N6 | 28°59'48.72"N | 113° 6'59.76"W |
| Viborita, Isla Angel de la Guarda, Baja California, Mexico | N6 | 29° 3'44.58"N | 113° 5'39.36"W |
| Isla Tiburón, Sonora, Mexico | N7 | 28°52'15.60"N | 112°33'51.96"W |
| Isla Tiburón, Sonora, Mexico | N7 | 28°52'57.42"N | 112°34'3.90"W |
| Isla Tiburón, Sonora, Mexico | N7 | 29° 0'22.73"N | 112°30'5.90"W |
| Isla Cholludo, Sonora, Mexico | N7 | 28°44'18.36"N | 112°18'18.06"W |
| Isla Datil, Sonora, Mexico | N7 | 28°43'50.12"N | 112°17'39.05"W |
| Isla San Esteban, Sonora, Mexico | N8 | 28°41'11.76"N | 112°32'45.24"W |
| San Rafael, Baja California, Mexico | N9 | 28°34'18.78"N | 113° 7'12.66"W |
| San Luis Gonzaga, Baja California, Mexico | N10 | 29°48'36.00"N | 114°23'34.86"W |
| San Luis Gonzaga, Baja California, Mexico | N10 | 29°47'43.32"N | 114°23'47.16"W |
| Puertecitos, Baja California, Mexico | N11 | 30°22'10.41"N | 114°38'29.76"W |
| San Felipe, Baja California, Mexico | N12 | 31° 1'31.97"N | 114°49'52.01"W |
| San Felipe, Baja California, Mexico | N12 | 31° 2'13.92"N | 114°49'31.44"W |
| La Cholla, Sonora, Mexico | N13 | 31°20'45.78"N | 113°38'12.42"W |
| Puerto Peñasco, Sonora, Mexico | N14 | 31°17'46.72"N | 113°32'34.02"W |
| Puerto Lobos, Sonora, Mexico | N15 | 30°15'52.44"N | 112°51'25.14"W |
| Puerto Libertad, Sonora, Mexico | N16 | 29°54'9.61"N | 112°43'36.31"W |
