# Supplementary material for: Phylogeography of Supralittoral Rocky Intertidal Ligia Isopods in the Pacific Region from Central California to Central Mexico
Source: PLoS One. 2010 Jul 21;5(7):e11633. doi: 10.1371/journal.pone.0011633 (PMC2908127; doi:10.1371/journal.pone.0011633)
Supplement: Table S2 — Ranges of Kimura-2-parameter distances observed among main Ligia clades found in our study area and outgroups. Upper matrix: COI gene distances. Lower matrix: 16S rDNA gene distances. Values on diagonal show maximum within-clade divergence (left: COI gene; right: 16S rDNA gene). (0.07 MB DOC) [file pone.0011633.s003.doc]

|  | Central California (A) | Southern California (B) | California (C) | Baja Pacific North (D) | Baja Pacific South (E) | Gulf South (S) | Gulf North (N) | Careyes (F) | *Ligia exotica* | *Ligia perkinsi* | *Ligia hawaiensis* | *Ligia vitiensis* | *Ligia pallasi* | *Ligia italica* | *Ligia oceanica* | *Ligidium hypnorum* |
| --- | --- | --- | --- | --- | --- | --- | --- | --- | --- | --- | --- | --- | --- | --- | --- | --- |
| Central California (A) | **1.55, 0.37** | 19.67–24.39 | 21.06–23.12 | 21.09–23.00 | 21.70–23.65 | 17.62–23.63 | 20.58–27.20 | 20.50–20.97 | 22.35–23.59 | 22.29–22.78 | 23.57–24.32 | 24.14–24.91 | 29.41–30.67 | 26.58–26.98 | 28.75–29.81 | 35.64–36.53 |
| Southern California (B) | 8.48–11.09 | **8.60^a^, 2.22** | 17.33–19.45 | 17.01–19.60 | 15.96–18.08 | 19.51–25.76 | 21.29–29.19 | 19.37–22.57 | 21.93–23.49 | 20.41–21.77 | 22.46–24.04 | 27.88–30.77 | 29.07–31.53 | 28.21–31.10 | 26.68–28.19 | 39.11–41.57 |
| California (C) | 5.39–6.89 | 3.69–6.39 | **2.10^b^, 0.00** | 7.28–8.16 | 13.27–15.46 | 19.49–25.34 | 20.14–29.89 | 20.29–21.40 | 20.18–21.31 | 19.72–21.29 | 22.14–23.57 | 22.65–23.41 | 30.06–31.32 | 26.56–27.35 | 26.16–27.17 | 39.64–41.08 |
| Baja Pacific North (D) | 5.75–6.89 | 3.76–6.36 | 0.00–0.00 | **2.08, 0.00** | 12.02–14.61 | 18.77–24.82 | 19.67–27.30 | 21.93–22.77 | 20.16–20.88 | 21.30–21.35 | 24.10–24.81 | 21.25–22.37 | 30.38–30.80 | 26.11–26.88 | 27.45–27.98 | 38.28–39.21 |
| Baja Pacific South (E) | 6.52–8.13 | 2.99–7.50 | 1.02–1.46 | 1.02–1.36 | **8.77^c^, 2.41** | 16.54–24.73 | 19.02–25.98 | 21.53–21.53 | 18.06–20.35 | 21.36–23.49 | 21.11–22.78 | 25.34–28.46 | 31.74–35.05 | 28.99–31.32 | 25.24–26.87 | 41.36–43.86 |
| Gulf South (S) | 8.86–12.90 | 3.36–8.59 | 5.24–8.95 | 6.35–8.64 | 5.60–8.25 | **21.55^d^, 5.63** | 15.16–26.47 | 17.59–23.56 | 21.18–25.43 | 21.24–25.61 | 22.06–26.31 | 25.41–29.03 | 26.22–31.63 | 25.02–29.43 | 26.01–31.71 | 31.44–37.63 |
| Gulf North (N) | 7.13–13.36 | 4.51–10.21 | 5.81–10.21 | 6.51–9.86 | 5.97–11.09 | 5.32–11.09 | **25.30^e^, 9.55** | 19.73–24.47 | 19.63–26.66 | 20.68–29.26 | 23.05–27.26 | 23.55–30.14 | 26.95–34.38 | 27.04–32.42 | 26.92–33.06 | 31.78–41.78 |
| Careyes (F) | 6.90–8.13 | 4.55–7.50 | 2.80–3.26 | 3.10–3.10 | 4.17–4.17 | 6.02–8.69 | 5.99–9.89 | **1.14, 0.00** | 19.80–20.02 | 18.03–18.46 | 20.02–20.02 | 25.92–26.33 | 25.45–25.83 | 23.93–25.06 | 24.80 | 35.29 |
| *Ligia exotica* | 7.34–7.92 | 5.35–7.11 | 4.48–5.41 | 5.26–5.26 | 5.26–6.38 | 7.11–9.03 | 7.48–9.47 | 6.76 | **6.84, 1.37** | 23.25–24.26 | 22.53–22.65 | 24.52–29.41 | 31.46–32.21 | 37.00 | 29.44–31.08 | 40.01–40.97 |
| *Ligia perkinsi* | 10.83–12.33 | 12.39–14.65 | 12.55–13.79 | 13.51–13.51 | 13.08–14.37 | 10.56–13.37 | 12.12–17.37 | 13.86 | 12.7–13.08 | na | 14.14 | 26.42 | 27.11 | 24.37 | 36.56 | 35.33 |
| *Ligia hawaiensis* | 11.19–12.69 | 11.49–14.13 | 11.21–13.2 | 12.14–12.14 | 11.73–12.56 | 10.48–12.07 | 11.26–14.22 | 12.51 | 10.2–10.56 | 5.6 | na | 26.95 | 27.47 | 27.74 | 25.47 | 36.56 |
| *Ligia vitiensis* | 14.14–15.49 | 15.65–18.19 | 13.08–14.69 | 14.69–14.69 | 14.25–15.12 | 16.44–17.64 | 14.86–19.97 | 15.05 | 16.52–16.91 | 12.17 | 11.73 | na | 29.07 | 28.92 | 33.63 | 36.04 |
| *Ligia pallasi* | 17.79–19.41 | 17.29–18.69 | 14.77–16.93 | 15.71–15.71 | 15.71–16.14 | 17.34–19.05 | 15.63–19.14 | 16.53 | 16.99–17.36 | 18.78 | 16.93 | 19.59 | na | 26.61 | 33.39 | 34.42 |
| *Ligia italica* | 18.22–18.46 | 14.62–16.77 | 14.33–15.89 | 14.83–14.83 | 15.25–16.1 | 17.74–20.42 | 16.89–19.11 | 17.8 | 16.89–17.83 | 19.62 | 19.09 | 23.88 | 18.32 | na | 31.60 | 34.42 |
| *Ligia oceanica* | 21.97–22.81 | 20.48–21.45 | 17.84–20.63 | 18.74–18.74 | 18.74–20.12 | 23.3–26.33 | 19.51–23.83 | 19.59 | 21.06–21.45 | 25.58 | 22.02 | 22.99 | 16.17 | 18.27 | na | 41.08 |
| *Ligidium hypnorum* | 19.67–19.99 | 23.54–24.56 | 21.27–22.5 | 21.27–21.27 | 20.73– 21.27 | 19.72–22.92 | 21.22–24.05 | 22.48 | 22.3–22.41 | 21.81 | 22.26 | 22.81 | 22.81 | 24.38 | 22.71 | na |

^a, b, c, d, e^ Divergences among lineages/localities within these clades are shown in Supporting Tables S3–S7.
