# Supplementary material for: Phylogeography of Supralittoral Rocky Intertidal Ligia Isopods in the Pacific Region from Central California to Central Mexico
Source: PLoS One. 2010 Jul 21;5(7):e11633. doi: 10.1371/journal.pone.0011633 (PMC2908127; doi:10.1371/journal.pone.0011633)
Supplement: Table S3 — Cytochrome Oxidase I (COI) gene percent divergence (Kimura-2-parameter correction) ranges within (diagonal) and among (below diagonal) selected lineages in the Southern California clade (B; green in Fig. 3). (0.04 MB DOC) [file pone.0011633.s004.doc]

|  | **IndusArea (B1), IthsmusC1 (B3)** | **LittleHa1 (B2)** | **RefugioB1 (B7)** | **LongBeach (B5)** |
| --- | --- | --- | --- | --- |
| **IndusArea (B1), IthsmusC1 (B3)** | **0.00** |  |  |  |
| **LittleHa1 (B2)** | 2.38 | **na** |  |  |
| **RefugioB1 (B7)** | 7.26 | 6.89 | **na** |  |
| **LongBeach (B5)** | 8.04 | 7.53 | 2.60 | **na** |
| **Sandiego (B6)** | 2.42 | 0.90 | 7.08 | 8.6 |
