# Supplementary material for: Phylogeography of Supralittoral Rocky Intertidal Ligia Isopods in the Pacific Region from Central California to Central Mexico
Source: PLoS One. 2010 Jul 21;5(7):e11633. doi: 10.1371/journal.pone.0011633 (PMC2908127; doi:10.1371/journal.pone.0011633)
Supplement: Table S4 — Cytochrome Oxidase I (COI) gene percent divergence (Kimura-2-parameter correction) among localities in the California 2 clade (C; orange in Fig. 3). (0.03 MB DOC) [file pone.0011633.s005.doc]

|  | **Corona En (C2)** | **Ford Point (C3)** | **E Point 26 (C4)** | **Johnsons L (C5)** | **S Frenchys 61 (C6)** | **Sandy B SR (C7)** |
| --- | --- | --- | --- | --- | --- | --- |
| **Ford Point (C3)** | 0.51 |  |  |  |  |  |
| **E Point 26 (C4)** | 1.03 | 0.51 |  |  |  |  |
| **Johnsons L (C5)** | 1.20 | 0.68 | 0.17 |  |  |  |
| **S French 61 (C6)** | 1.03 | 0.51 | 0.00 | 0.17 |  |  |
| **Sandy B SR (C7)** | 0.68 | 0.17 | 0.68 | 0.85 | 0.68 |  |
| **San Diego (C1)** | 1.22 | 1.75 | 1.93 | 2.10 | 1.93 | 1.93 |
