# Supplementary material for: Phylogeography of Supralittoral Rocky Intertidal Ligia Isopods in the Pacific Region from Central California to Central Mexico
Source: PLoS One. 2010 Jul 21;5(7):e11633. doi: 10.1371/journal.pone.0011633 (PMC2908127; doi:10.1371/journal.pone.0011633)
Supplement: Table S5 — Cytochrome Oxidase I (COI) gene percent divergence (Kimura-2-parameter correction) ranges within (diagonal) and among (below diagonal) selected groups of localities in the Baja Pacific South clade (BPS; turquoise in Fig. 3). (0.05 MB DOCX) [file pone.0011633.s006.docx]

|  | **BAsuncion (E5), SanHipo2 (E6)** | **Cedros37, Cedros36 (E1)** | **PSCarlos (E8)** | **PEugenia (E2), ElChevo (E3), Tortugas (E4)** | **ElQueen, Malarrimo (E3)** |
| --- | --- | --- | --- | --- | --- |
| **BAsuncion (E5), SanHipo2 (E6)** | **0.51–0.51** |  |  |  |  |
| **Cedros37, Cedros36 (E1)** | 6.64–6.82 | **0.00** |  |  |  |
| **PSCarlos (E8)** | 8.76–8.77 | 3.13–3.13 | **na** |  |  |
| **PEugenia (E2), ElChevo (E3), Tortugas (E4)** | 7.98–8.19 | 1.90–2.07 | 2.60–2.96 | **0.17–0.51** |  |
| **ElQueen, Malarrimo (E3)** | 7.59–8.19 | 2.25–2.25 | 2.96–3.32 | 1.03–1.20 | **0.34** |
