# Supplementary material for: Phylogeography of Supralittoral Rocky Intertidal Ligia Isopods in the Pacific Region from Central California to Central Mexico
Source: PLoS One. 2010 Jul 21;5(7):e11633. doi: 10.1371/journal.pone.0011633 (PMC2908127; doi:10.1371/journal.pone.0011633)
Supplement: Table S6 — Cytochrome Oxidase I (COI) gene percent divergence (Kimura-2-parameter correction) ranges within (diagonal) and among (below diagonal) selected groups of localities in the Gulf North clade (red in Figs. 3 and 4). Shapes refer to clades defined in Fig. 4. (0.05 MB DOC) [file pone.0011633.s007.doc]

|  |  | squares | | | pentagons | | | circles | | | triangle |
| --- | --- | --- | --- | --- | --- | --- | --- | --- | --- | --- | --- |
|  |  | SRosalia2 (N2) San Bruno (N1) | SPedroMar (N3) | SFrancisq (N4) | Tiburon Idatil Cholludo (N7) | SRafaelce (N9) | SEsteban (N8) | PLobos (N15) PLibertaD (N16) | Puertecitos (N11) Cholla (N13) PPenasco (N14) SanFelipe (N12) | SLGonzaga (N10) | Angel de la Guarda (N6) |
| squares | SRosalia2 (N2) San Bruno (N1) | **0.49-0.53** |  |  |  |  |  |  |  |  |  |
| pentagons | SPedroMar (N3) | 14.80–15.12 | **na** |  |  |  |  |  |  |  |  |
|  | SFrancisq (N4) | 10.97–11.40 | 15.46 | **na** |  |  |  |  |  |  |  |
|  | Tiburon Idatil Cholludo (N7) | 16.29–18.64 | 16.00–22.76 | 18.47-19.09 | **0.17–1.05** |  |  |  |  |  |  |
| circles | SRafaelce (N9) | 16.62–18.34 | 18.70 | 17.94 | 17.07–17.70 | **na** |  |  |  |  |  |
|  | SEsteban (N8) | 16.08–17.61 | 18.90 | 17.39 | 15.91–16.82 | 17.07 | **na** |  |  |  |  |
|  | PLobos (N15) PLibertaD (N16) | 17.40–18.16 | 22.24–22.51 | 20.09–20.57 | 20.11–21.14 | 22.87–24.06 | 22.21–22.48 | **1.47** |  |  |  |
|  | Puertecitos (N11) Cholla (N13) PPenasco (N14) SanFelipe (N12) | 19.99–21.45 | 22.58–23.35 | 24.52–25.27 | 22.16–24.31 | 21.58–22.52 | 21.77–22.72 | 17.15–19.07 | **0.49–1.31** |  |  |
|  | SLGonzaga (N10) | 21.77–22.30 | 24.37 | 25.30 | 23.17–24.35 | 24.18 | 23.17 | 17.78–18.42 | 4.38–4.73 | **na** |  |
| triangle | Angel de la Guarda (N6) | 17.46–18.19 | 18.37–18.46 | 19.89–20.34 | 17.57–18.57 | 20.36–21.06 | 18.75–19.35 | 18.37–18.70 | 18.68–19.76 | 20.37–20.93 | **na** |
| X | Bahia de los Angeles (N5) | 16.34–16.79 | 19.66 | 18.00 | 17.02–17.90 | 20.02 | 20.63 | 17.23–18.16 | 19.50–19.97 | 21.14 | 16.79–17.13 |
