# Supplementary material for: Phylogeography of Supralittoral Rocky Intertidal Ligia Isopods in the Pacific Region from Central California to Central Mexico
Source: PLoS One. 2010 Jul 21;5(7):e11633. doi: 10.1371/journal.pone.0011633 (PMC2908127; doi:10.1371/journal.pone.0011633)
Supplement: Table S7 — Cytochrome Oxidase I (COI) gene percent divergence (Kimura-2-parameter correction) ranges within (diagonal) and among (below diagonal) selected groups of localities in the Gulf South clade (blue in Figure 4). Shapes refer to clades defined in Fig. 4. (0.08 MB DOC) [file pone.0011633.s008.doc]

|  |  | Stars | | | Hexagons | | | Squares | | Diamond | X | Circles | | | Triangles | | |
| --- | --- | --- | --- | --- | --- | --- | --- | --- | --- | --- | --- | --- | --- | --- | --- | --- | --- |
|  |  | Cajete, Evaristo (S2) | Loretosur (S1) | SBrunito (S3), SanCosme (S4) | PSueno1, BConcepN (S6), Pchivato, Mulege (S5) | Bventura , Armenta, Requeson, BConcepcS (S6) | SRosalia, SLucas (S7) | Kino (S8), SanCarlos (S9), Guaymas1 (S10) | SNicolas (S27) | IPartida (S12), ESanto60 (S12), LaPaz (S11), ESanto61 (S12) | CaboPulmo (S26) | PMita (S21), SanBlas (S23), MazaCerri (S24), Platan22 (S23), MazatUNAM (S24), Aticama (S23), ICoral1 (S22) | Manzanill (S20), Ixtapa (S18), BPotosi (S17), Boquita (S20), Zihuatane (S18), Carrizali (S19) | Topolobam (S25) | Barriles, EMuertos (S14), Frailes (S15) | Cerral68,Cerral66, Cerral67 (S13) | SLElArco, CaboSanLu (S16) |
| Stars | Cajete, Evaristo (S2) | **0.16** |  |  |  |  |  |  |  |  |  |  |  |  |  |  |  |
|  | Loretosur (S1) | 4.39–4.56 | **na** |  |  |  |  |  |  |  |  |  |  |  |  |  |  |
|  | SBrunito (S3), SanCosme (S4) | 3.17––3.71 | 3.17–3.71 | **0.54** |  |  |  |  |  |  |  |  |  |  |  |  |  |
| Hexagons | PSueno1, BConcepN (S6), Pchivato, Mulege (S5) | 14.70–15.34 | 14.25–14.50 | 15.15–15.99 | **0.00–0.53** |  |  |  |  |  |  |  |  |  |  |  |  |
|  | Bventura , Armenta, Requeson, BConcepcS (S6) | 16.28–16.52 | 16.07–16.09 | 16.07–16.44 | 2.31–2.87 | **0.16–0.65** |  |  |  |  |  |  |  |  |  |  |  |
|  | SRosalia, SLucas (S7) | 15.64–15.86 | 16.50–16.50 | 16.71–16.90 | 7.43–7.57 | 7.95–8.50 | **0.00** |  |  |  |  |  |  |  |  |  |  |
| Squares | Kino (S8), SanCarlos (S9), Guaymas1 (S10) | 14.04–14.66 | 13.63–14.04 | 13.43–14.59 | 15.58–16.64 | 15.98–17.03 | 16.21–16.64 | **0.32** |  |  |  |  |  |  |  |  |  |
|  | SNicolas (S27) | 14.98–15.22 | 14.50–14.50 | 14.74–14.87 | 15.86–16.42 | 17.12–17.34 | 16.15–16.15 | 8.55–8.98 | **na** |  |  |  |  |  |  |  |  |
| Diamond | IPartida (S12), ESanto60 (S12), LaPaz (S11), ESanto61 (S12) | 14.70–16.23 | 14.07–15.05 | 14.28–15.28 | 19.03–20.26 | 18.72–20.00 | 20.06–21.55 | 13.25–14.36 | 12.48–13.04 | **0.00–0.18** |  |  |  |  |  |  |  |
| X | CaboPulmo (S26) | 14.15–14.36 | 13.52–13.52 | 13.11–13.33 | 19.62–20.06 | 19.15–19.60 | 18.70–18.70 | 13.08–13.49 | 15.61–15.61 | 12.61–13.42 | **na** |  |  |  |  |  |  |
| Circles | PMita (S21), SanBlas (S23), MazaCerri (S24), Platan22 (S23), MazatUNAM (S24), Aticama (S23), ICoral1 (S22) | 13.36–14.89 | 14.51–15.74 | 13.82–15.77 | 17.62–19.64 | 17.52–19.30 | 16.02–16.87 | 12.03–13.07 | 12.91–13.85 | 10.97–13.37 | 10.44–11.65 | **0.00–2.01** |  |  |  |  |  |
|  | Manzanill (S20), Ixtapa (S18), BPotosi (S17), Boquita (S20), Zihuatane (S18), Carrizali (S19) | 14.00–14.58 | 15.10–15.64 | 14.47–15.77 | 18.16–19.29 | 17.28–18.18 | 16.84–17.06 | 12.97–13.75 | 14.28–14.52 | 12.61–14.23 | 12.15–12.51 | 3.36–4.59 | 0**.00–0.82** |  |  |  |  |
|  | Topolobam (S25) | 15.10–15.31 | 16.60–16.60 | 15.53–16.49 | 18.51–19.29 | 17.96–18.63 | 17.52–17.52 | 13.07–13.27 | 14.08–14.08 | 11.95–12.92 | 11.85–11.85 | 1.81–2.48 | 2.50–3.02 | **na** |  |  |  |
| Triangles | Barriles, EMuertos (S14), Frailes (S15) | 14.87–15.29 | 14.67–15.08 | 15.29–15.77 | 19.30–20.17 | 19.48–20.32 | 17.25–17.46 | 12.98–13.40 | 14.26–14.50 | 14.49–15.78 | 11.50–11.70 | 10.91–12.06 | 11.46–11.81 | 11.69–12.06 | **0.16–0.33** |  |  |
|  | Cerral68,Cerral66, Cerral67 (S13) | 13.24–14.03 | 13.24–13.81 | 13.80–14.40 | 19.38–20.50 | 19.38–20.67 | 17.51–18.12 | 13.73–14.63 | 14.01–14.27 | 14.33–15.33 | 11.11–11.47 | 9.90–11.38 | 10.83–11.80 | 11.43–11.80 | 2.62–3.01 | **0.18–0.51** |  |
|  | SLElArco, CaboSanLu (S16) | 14.51–14.93 | 14.30–14.51 | 14.09–15.35 | 18.96–19.98 | 18.90–19.58 | 17.78–18.70 | 12.26–12.46 | 16.05–16.30 | 11.95–13.35 | 10.62–10.82 | 9.54–10.60 | 9.63–10.48 | 10.02–10.21 | 7.38–7.86 | 7.45–7.84 | **0.02** |
